# In Vitro Tissue Reconstruction Using Decellularized Pericardium Cultured with Cells for Ligament Regeneration

**DOI:** 10.3390/polym14122351

**Published:** 2022-06-10

**Authors:** Mika Suzuki, Tsuyoshi Kimura, Yukina Yoshida, Mako Kobayashi, Yoshihide Hashimoto, Hironobu Takahashi, Tatsuya Shimizu, Shota Anzai, Naoko Nakamura, Akio Kishida

**Affiliations:** 1 Department of Material-Based Medical Engineering, Institute of Biomaterials and Bioengineering, Tokyo Medical and Dental University, Tokyo 101-0062, Japan; mika.mbme@tmd.ac.jp (M.S.); ma200041@tmd.ac.jp (Y.Y.); mako.mbme@tmd.ac.jp (M.K.); hashimoto.atrm@tmd.ac.jp (Y.H.); naoko@shibaura-it.ac.jp (N.N.); kishida.mbme@tmd.ac.jp (A.K.); 2 Institute of Advanced Biomedical Engineering and Science, Tokyo Women’s Medical University, Tokyo 162-8666, Japan; takahashi.hironobu@twmu.ac.jp (H.T.); shimizu.tatsuya@twmu.ac.jp (T.S.); 3 Department of Bioscience and Engineering, Shibaura Institute of Technology, Saitama 337-8570, Japan; bn16228@shibaura-it.ac.jp

**Keywords:** decellularization, extracellular matrix, porcine pericardium, high hydrostatic pressurization method, surfactant method, 3D fabrication, ligament

## Abstract

Recent applications of decellularized tissues have included the ectopic use of their sheets and powders for three-dimensional (3D) tissue reconstruction. Decellularized tissues are fabricated with the desired functions to employ them to a target tissue. The aim of this study was to develop a 3D reconstruction method using a recellularized pericardium to overcome the difficulties in cell infiltration into tight and dense tissues, such as ligament and tendon tissues. Decellularized pericardial tissues were prepared using the high hydrostatic pressurization (HHP) and surfactant methods. The pericardium consisted of bundles of aligned fibers. The bundles were slightly disordered in the surfactant decellularization method compared to the HHP decellularization method. The mechanical properties of the pericardium were maintained after the HHP and surfactant decellularizations. The HHP-decellularized pericardium was rolled up into a cylindrical formation. Its mechanical behavior was similar to that of a porcine anterior cruciate ligament in tensile testing. NIH3T3, C2C12, and mesenchymal stem cells were adhered with elongation and alignment on the HHP- and surfactant-decellularized pericardia, with dependences on the cell type and decellularization method. When the recellularized pericardium was rolled up into a cylinder formation and cultured by hanging circulation for 2 days, the cylinder formation and cellular elongation and alignment were maintained on the decellularized pericardium, resulting in a layer structure of cells in a cross-section. According to these results, the 3D-reconstructed decellularized pericardium with cells has the potential to be an attractive alternative to living tissues, such as ligament and tendon tissues.

## 1. Introduction

Tissue engineering and regenerative medicine are emerging fields focused on curing complex and chronic diseases. Decellularized tissue prepared from a living tissue is an extracellular matrix, which can be used as a scaffold in tissue engineering, owing to its low immunogenicity and high compatibility. Numerous decellularized tissues, such as the dermis [1], urinary bladder matrix [2], small intestinal submucosa [3], aorta [4,5], ligament, and tendon [6,7,8], have been developed using various physical and chemical decellularization methods. Mainly, decellularized tissues have been employed as alternative tissues. Particularly, for tissues where mechanical compliance is important, such as vascular, ligament, and tendon tissues, decellularized tissues are implanted orthotopically [9] by retaining the histological structure to express typical biomechanical properties. Numerous decellularization methods have been developed to remove cells while maintaining the tissue structure [10,11]. Although decellularization has been achieved effectively for tight, rigid, and dense tissues such as ligament, tendon, and vascular tissues, recipient cells cannot easily infiltrate these tissues [8,12]. Moreover, complications such as a repair-site rupture and adhesion formation slow the healing because of the hypocellular composition of tissues [13]. Thus, decellularized tissues are fabricated by creating holes and slits to promote cell infiltration while maintaining their shape [12]. Decellularized tissues have been formed into powders [14], sheets [15], and gels [16] for ectopic use and three-dimensional (3D) printing [17]. Several decellularized tissues are commercially available and used ectopically, e.g., for wound healing and adhesion prevention [18,19]. We reported that a sheet of decellularized aortic intermedia was formed into a tube and applied as an alternative small-diameter vascular graft [20,21].

**Figure 1:**
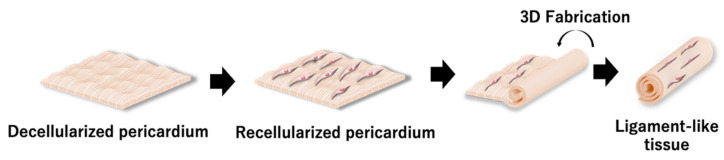
Scheme of a 3D tissue reconstructed using the recellularized pericardium.

In this study, we propose a 3D tissue fabrication method using a decellularized membranous tissue recellularized to apply it as a ligament and tendon alternative. Decellularized pericardia were prepared by physical and chemical decellularization methods as decellularized membranous tissues, and then were recellularized by several types of cells: fibroblast, myoblast, and mesenchymal stem cells. The recellularized pericardium was rolled up, formed cylindrically, and cultured in vitro as a reconstructed ligament-like tissue (Figure 1).

## 2. Materials and Methods

### 2.1. Materials

Fresh porcine pericardium and anterior cruciate ligament (ACL) were obtained from a local slaughterhouse (Tokyo Shibaura Zouki, Tokyo, Japan) and stored at 4 °C until use. DNase I was purchased from Roche Diagnostics (Tokyo, Japan). Dulbecco’s modified Eagle medium (DMEM), magnesium chloride hexahydrate (MgCl_2_), neutral-buffered (pH = 7.4) solution of 10% formalin, protease-K, phosphate-buffered saline (PBS), NaCl, sodium deoxycholate (SDC), sodium dodecyl sulfate (SDS), and tert-butyl alcohol were purchased from FUJIFILM Wako Pure Chemical Corp. (Osaka, Japan). Bovine serum albumin and tris(hydroxymethyl)aminomethane (Tris) were obtained from Sigma-Aldrich Inc., (St. Louis, MO, USA). An isodine solution was purchased from Shionogi Healthcare (Osaka, Japan). Gentamicin sulfate was procured from Tokyo Chemical Industry Co. Ltd. (Tokyo, Japan). Glutaraldehyde (25%) was obtained from TAAB Laboratories Equipment, Ltd. (Aldermaston, UK). Phenol/chloroform was purchased from Nippon Gene (Tokyo, Japan). Ethylenediaminetetraacetic acid (EDTA) and Calcein-AM solution (0.5 mg/mL) were purchased from Dojindo Laboratories (Kumamoto, Japan). NIH3T3 and C2C12 cells were purchased from Riken Bioresource Research Center (Tsukuba, Japan). Mesenchymal stem cells were purchased from JCRB Cell Bank, National Institute of Biomedical Innovation, Health and Nutrition (Osaka, Japan). A mesenchymal stem cell medium (Powered by 10) was purchased from GlycoTechnica Ltd. (Yokohama, Japan).

### 2.2. Preparation of a Decellularized Porcine Pericardium

The porcine pericardium was separated into a fibrous pericardium and serous pericardium. The serous pericardium was trimmed by removing fat off the underside of the parietal pericardium. The pericardium was packed into a plastic bag containing saline and sealed. The pericardium was subjected to a high hydrostatic pressure of 1000 MPa at 30 °C for 10 min using a high-hydrostatic-pressure machine (Dr. Chef; Kobelco, Kobe, Japan), and then washed with DNase (0.1 mg/mL) and MgCl_2_ (50 mM) in saline at 4 °C for 4 days, 80% ethanol in saline at 4 °C for 3 days, and 0.1 M citric acid in a saline solution at 4 °C for 3 days. Surfactant decellularization was carried out according to reported procedures [22]. The pericardium was treated with a solution of 0.5% SDS and 0.5% SDC for 24 h, and then washed with PBS at room temperature for 12 h six times.

### 2.3. Deoxyribonucleic Acid (DNA) Quantification

Residual DNA quantification was performed to evaluate the quality of the decellularization. The decellularized pericardium was freeze-dried and incubated in a lysis buffer containing 50-mgmL^−1^ protease K, 50 mM Tris–HCl, 1% SDS, 10 mM NaCl, and 20 mM EDTA at 55 °C for 12 h. The DNA was purified with phenol/chloroform extraction and ethanol precipitation. The residual DNA content was quantified at 260 nm using a microvolume spectrophotometer (Nanodrop; Thermo Fisher Scientific K.K., Tokyo, Japan) and normalized to the tissue dry weight of 20 mg.

### 2.4. Hematoxylin–Eosin (H&E) Staining

To evaluate the efficiency of cell removal, decellularized pericardia were fixed with a neutral-buffered (pH = 7.4) solution of 10% formalin in PBS at 25 °C for 24 h and dehydrated with 70%, 80%, 90%, and 100% ethanol. The samples were then replaced with xylene and embedded in paraffin. Paraffin sections were cut into 4 µm thick sections and stained with hematoxylin and eosin.

### 2.5. Scanning Electron Microscopy (SEM) Observation of the Pericardium

The decellularized pericardia were fixed for 24 h at room temperature in 2.5% glutaraldehyde, dehydrated through a graded ethanol series, soaked for 72 h in t-butyl alcohol, sputter-coated with Au, and imaged with SEM (S-3400N, Hitachi Ltd., Tokyo, Japan).

### 2.6. Mechanical Properties

The mechanical strengths of the untreated pericardium and high-hydrostatic-pressurization (HHP)- and surfactant-decellularized pericardia were evaluated. All samples were cut into dumbbell-shaped pieces. The test pieces were 35 mm long and 2 mm wide. Wall thicknesses were measured using a creep meter (RE2-33005 B, Yamaden Co., Ltd., Tokyo, Japan) for each sample. The HHP-decellularized pericardium (5 cm × 13 cm) was tightly rolled up into a cylinder form. Stress–strain curves of the pieces and cylinder of the pericardium were obtained using a universal testing machine (AGS-X, Shimadzu Co., Ltd., Kyoto, Japan). Each sample was strained at a rate of 5 mm/min.

### 2.7. Cell Culture

DMEM containing 10% FBS and 1% penicillin/streptomycin was used for cell culture of C2C12 and NIH3T3. Poweredby 10 was used for cell culture of human mesenchymal stem cells (hMSCs). C2C12, NIH3T3, and hMSCs were seeded on the HHP- and surfactant-decellularized pericardia (2 × 10^4^ cells/cm^2^) and incubated at 37 °C under 5% CO_2_ for 4 days. The culture medium was changed every 2 days. After 2 and 4 days, the cells were incubated with Calcein-AM (1 µg/mL) at 37 °C for 30 min and observed using a fluorescent microscope (BZ-X710, Keyence Corp., Osaka, Japan) after washing with PBS twice. The cell growth was assessed by a cell counting kit-8 (Dojindo Labolratories, Kumamoto, Japan). After culturing for 1, 2, 3, and 4 days, the absorbance of WST-8 at 450 nm was measured with a multispectrometer (Cytation, BioTek Japan, Tokyo, Japan). The cell density was calculated using the standard curve of absorbance.

### 2.8. Cell Culture in the Cylindrically Formed Pericardium

A silicone mold (5 cm × 6.5 cm) with an empty space (3 cm × 4 cm) was placed on the HHP-decellularized pericardium (5 cm × 7 cm). The C2C12 cells were seeded on the HHP-decellularized pericardium at a cell density of 2 × 10^4^ cells/cm^2^ and cultured for 1 day. A medical-grade suture was placed on one end of the recellularized pericardium. The pericardium was rolled up into a cylinder form so that its suture was in the center. Both ends of the suture were fixed at the edge of the plastic cup and the rolled pericardium was hanged. After filling it with the culture medium, the rolled pericardium was cultured for 2 days with a stirring culture medium. After cutting it in half, one half was subjected to H&E staining. The other half was partially peeled, incubated with Calcein-AM (1 µg/mL) at 37 °C for 30 min, and observed using a fluorescent microscope after washing with PBS twice to evaluate the cell adhesion and survival.

### 2.9. Statistical Analysis

Each experiment was performed six times. The results are expressed as mean ± standard deviation. One-way analysis of variance (ANOVA) and Tukey’s post hoc multiple comparison tests were carried out to evaluate statistical significance. A *p*-value < 0.05 was considered statistically significant. For the cell growth analysis, the samples were compared each day.

## 3. Results

### 3.1. Preparation of the Decellularized Pericardium

**Figure 2:**
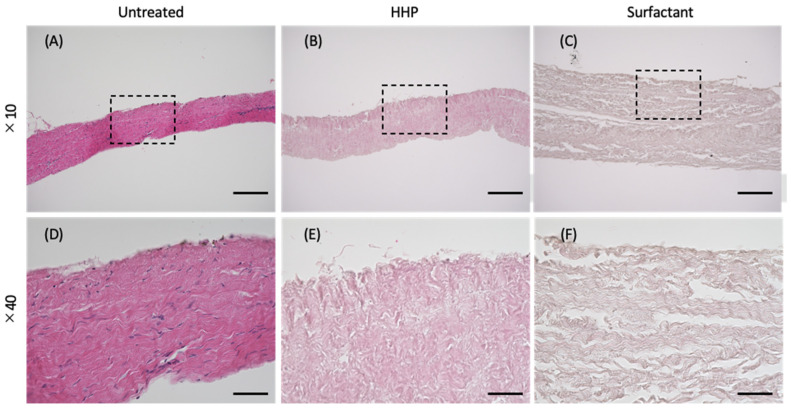
H&E staining of the (**A**,**D**) untreated, (**B**,**E**) HHP-decellularized, and (**C**,**F**) surfactant-decellularized pericardia. Scale bars: (**A**–**C**) 200 µm, (**D**–**F**) 50 µm. Nuclei can be observed in black in images (**A**,**D**), but are absent after the decellularization (**B**,**C**,**E**,**F**).

The porcine pericardia were decellularized with HHP and surfactant methods. Figure 2A–F show the H&E staining of the untreated pericardium, HHP-decellularized pericardium, and surfactant-decellularized pericardium. There were no large changes in the shape and thickness of the pericardium upon the decellularization. For the HHP- and surfactant-decellularized pericardia, no blue spots of cells were observed. Gaps between the bundles of fibers occurred in the HHP-decellularized pericardium, which were expanded in the surfactant-decellularized pericardium. The residual DNA was 1052.9 ± 61.6 ng/mg of tissue weight and significantly decreased to 33.1 ± 0.8 and 46.6 ± 0.9 ng/mg of tissue weight for the HHP and surfactant pericardia, respectively.

**Figure 3:**
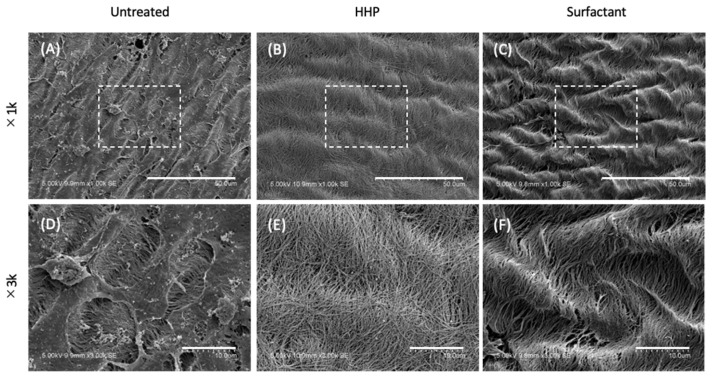
SEM observation of the (**A**,**D**) untreated, (**B**,**E**) HHP-decellularized, and (**C**,**F**) surfactant-decellularized pericardia. Scale bars: (**A**–**C**) 50 µm, (**D**–**F**) 10 µm.

Figure 3A–F show SEM images of the surfaces of the untreated pericardium, HHP-decellularized pericardium, and surfactant-decellularized pericardium. Adhered and spread cells were observed on the untreated pericardium. In gaps between the cells, aligned fibers were also observed. On the other hand, for the HHP-decellularized and surfactant-decellularized pericardia, no cells were observed, while wavy fibers were observed along the longitudinal direction of the fibers. In addition, melted and disordered fibers were partially observed for the surfactant-decellularized pericardium.

### 3.2. Mechanical Properties of the Decellularized Pericardium and 3D Reconstruction

**Figure 4:**
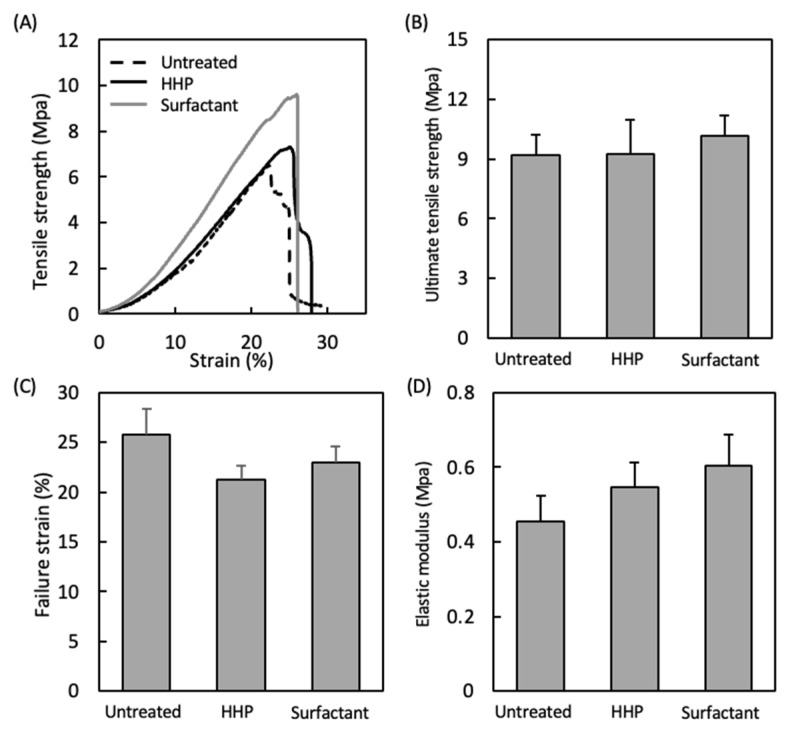
(**A**) Typical stress–strain curves of the untreated pericardium, HHP-decellularized pericardium, and surfactant-decellularized pericardium. Mechanical properties of the untreated pericardium, HHP-decellularized pericardium, and surfactant-decellularized pericardium: (**B**) ultimate tensile strength, (**C**) failure strain, and (**D**) elastic modulus. Mean ± standard deviation, *n* = 6; no significant differences were identified between groups (one-way ANOVA, *p* < 0.05; Tukey’s post hoc comparison).

Figure 4 shows the mechanical properties of the untreated pericardium and pericardia decellularized by the HHP and surfactant methods. Their stress–strain curves were J-curves, typical for biological tissues. There were no large differences in the mechanical parameters, such as the ultimate tensile strength, failure strain, and elastic modulus.

**Figure 5:**
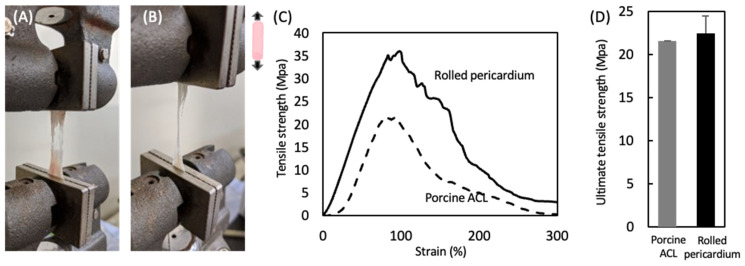
Photographs of the (**A**) porcine ACL and (**B**) rolled pericardium decellularized with the HHP method during a tensile test. Mechanical properties of the porcine ACL and rolled pericardium decellularized with the HHP method: (**C**) typical *S–S* curves and (**D**) ultimate tensile strength. Mean ± standard deviation, *n* = 6; no significant differences were identified between groups (one-way ANOVA, *p* < 0.05; Tukey’s post hoc comparison).

The cylindrically formed pericardium decellularized by the HHP method was subjected to a tensile test. Porcine ACL was used as a control. The porcine ACL and rolled pericardium were stretched, partially ruptured, and broken during the tensile test (Figure 5A,B). The typical stress–strain curves are shown in Figure 5C. The J-curve was observed in the early phase of the porcine ACL, and then the tensile strength was decreased slowly after the maximum tensile strength was reached, where the porcine ACL was partially ruptured. On the other hand, for the rolled pericardium, the shape of the S–S curve was similar to that of the porcine ACL, although the tensile strength increased without the J-shape in the early phase. There was no difference between their ultimate tensile strengths.

### 3.3. Recellularization of the Decellularized Pericardium and Hanging Circulation Culture of the 3D-Reconstructed Pericardium

**Figure 6:**
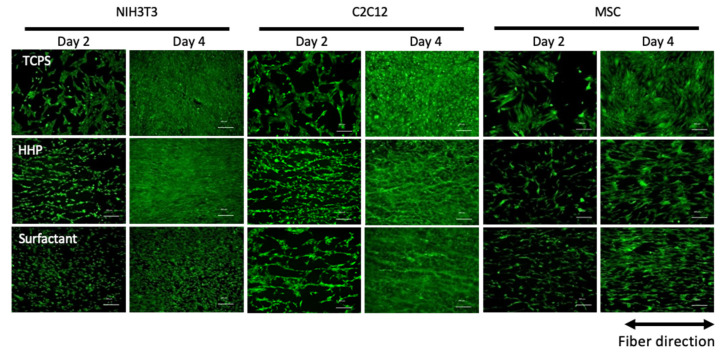
Fluorescent microscopy observation of various types of cells seeded on TCPS, HHP-decellularized pericardium, and surfactant-decellularized pericardium. The cells were stained with Calcein-AM, which shows living cells. Scale bar: 200 µm.

**Figure 7:**
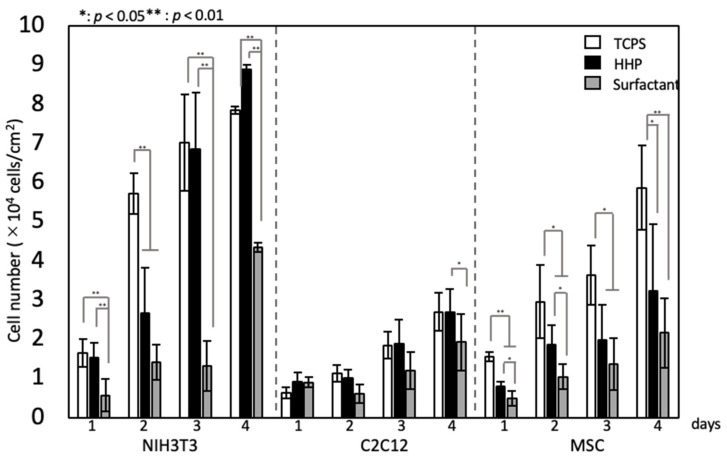
Cell growth on TCPS, HHP-decellularized pericardium, and surfactant-decellularized pericardium. Mean ± standard deviation, *n* = 6. One-way ANOVA, *: *p* < 0.05, **: *p* < 0.01; Tukey’s post hoc comparison.

Figure 6 shows fluorescent microscopy images of various types of cells seeded on the HHP- and surfactant-decellularized pericardia. TCPS was used as a control. All types of cells were adhered in random directions on the TCPS at day 2 and grew. On the other hand, on the HHP- and surfactant-decellularized pericardia, the cells were aligned along the fiber direction at day 2 and grew while maintaining the cellular direction for 4 days of cultivation. Elongated cells, whose longitudinal axis was parallel with the fiber direction, were observed on the HHP- and surfactant-decellularized pericardia at day 2 and kept for 4 days of culture. There were no large differences in the alignment and morphology of cells between the HHP- and surfactant-decellularized pericardia, although the cellular densities differed between them for NIH3T3 cells and MSCs. The cellular growth is shown in Figure 7. For the NIH3T3 cells, the growth was inhibited on the surfactant-decellularized pericardium, while an effective growth was observed on the HHP-decellularized pericardium. For the C2C12 cells, a slow growth was observed, irrespective of the substrate. For the MSCs, compared to TCPS, the cellular growth was suppressed on the HHP- and surfactant-decellularized pericardia.

**Figure 8:**
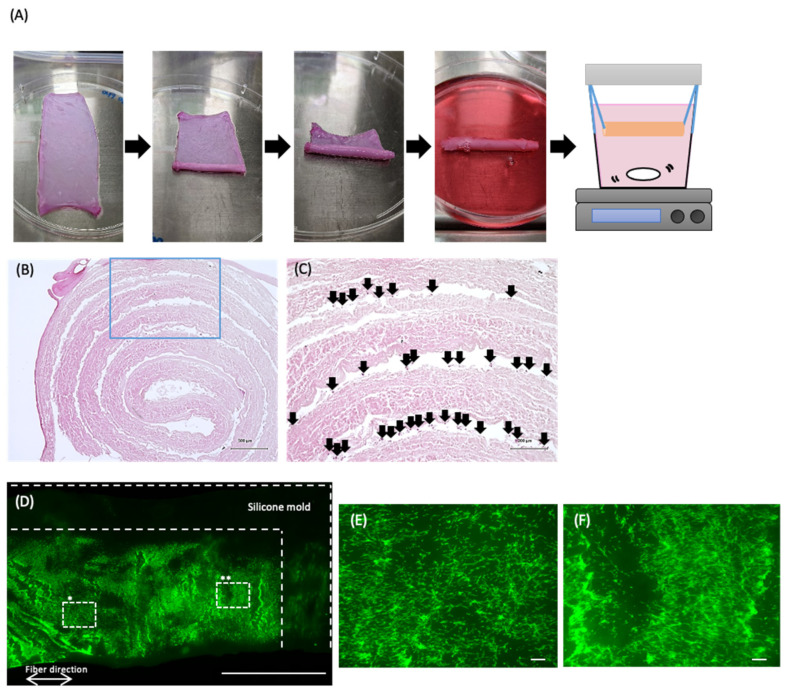
(**A**) Cylinder formation of the recellularized pericardium with C2C12 cells at a cell density of 2 × 10^3^ cells/cm^2^ and hanging culture. (**B**,**C**) H&E staining of the cross-section of the hanging-cultured pericardium for 2 days. The arrows show cells. (**D**–**F**) Fluorescent images of the Calcein-AM-stained cells on the partially peeled pericardium from the cylindrically formed pericardium after hanging culturing. (**E**,**F**): * and ** of hash square regions. Scale bars: (**D**) 1 cm, (**E**,**F**) 200 µm.

The HHP-decellularized pericardium was recellularized with C2C12 cells, formed cylindrically, and then hanging-cultured (Figure 8A). After 2 days of culture, the cylinder formation was kept. The cross-section of the cylindrically formed pericardium stained with H&E is shown in Figure 8B,C. The layer of the pericardia and numerous cells between layers were observed. Fluorescent microscopy images of cells stained with Calcein-AM, which can stain living cells, are shown in Figure 8D–F. The living cells were observed on the entire pericardium, although the cellular density varied with the location (Figure 8D). The living cells were oriented along the fiber direction (Figure 8E,F).

## 4. Discussion

Decellularized tissues are applied as alternative tissues. Numerous decellularization methods have been developed and an effective decellularization method with retained mechanical properties has been achieved. However, for tight and dense tissues, such as ligament, tendon, and vascular tissues, cells cannot easily infiltrate them. Recently, decellularized tissues have been fabricated into sheet and powder forms. They are used ectopically and induce recipient cells early when a mechanical contribution is not required [23]. We previously reported that the aortic intima-medium was formed into a tube and applied as a small-diameter vascular graft, which indicated that the mechanical compliance was adjusted by the appropriate fabrication of the decellularized tissue [20,21]. In this study, we developed a 3D tissue reconstruction of a ligament-like tissue with cells inside and similar mechanical properties to those of native tissues through the recellularization and fabrication of a decellularized pericardium.

Decellularized pericardia were prepared by the HHP and surfactant methods. The decellularization was achieved effectively while maintaining the mechanical properties, irrespective of the decellularization method. We reported that HHP decellularization could decellularize various tissues while retaining the tissue structure and mechanical properties [9]. On the other hand, the surfactant decellularization was an effective method, while the strong detergent destroyed the tissue structure, which contributed to the typical biological mechanical properties. We also reported that the SDS decellularization of the intima-media of aorta induced histological disordering and remarkable decreases in ultimate tensile strength and elastic modulus from 4.0 ± 1.9 to 1.5 ± 0.4 MPa and from 6.0 ± 2.9 to 2.4 ± 0.4 MPa, respectively [24]. However, the surfactant-decellularized pericardium was obtained with retained mechanical properties (Figure 4). It is considered that the combination of SDS and SDC could decellularize the pericardium [25]. A cylindrically formed pericardium was obtained, which exhibited a behavior similar to that of the porcine ACL in tensile testing (Figure 5D). This suggests that the appropriate fabrication could fit a target tissue even for the ectopic use of the cardiovascular tissue to a musculoskeletal tissue from a mechanics perspective. On the other hand, the ultimate tensile strength of a natural human ACL is approximately 36 MPa [26]. Thus, it is required to improve the rolling-up of the decellularized pericardium (e.g., number and strength) to apply it clinically.

The recellularization of various types of cells, NIH3T3, C2C12, and MSCs, was investigated in HHP- and surfactant-decellularized pericardia. Cellular adhesion and growth were shown on both decellularized pericardia. All types of cells were elongated and aligned on both decellularized pericardia (Figure 6). The percentages of NIH3T3, C2C12, and hMSCs with angles below 30° against the fiber direction were approximately 70%, 74%, and 49%, respectively, higher than those on TCPS (approximately 33%, 35%, and 33%, respectively) (data not shown). The cellular shape and alignment depend on the physical, chemical, and morphological properties of the substrate [27]. Particularly, the large elongation and alignment of cells were observed on the aligned fibers with nanoscale diameters compared to those with microscale diameters [28]. The SEM observation revealed that the surfaces of the HHP- and surfactant-decellularized pericardia consisted of a bundle of nanoscale fibers (71 ± 18 nm). The fiber bundles were aligned (Figure 3B,C,E,F). Thus, it is considered that the aligned fibrillar structures of the HHP- and surfactant-decellularized pericardia affected the cellular elongation and alignment along the fiber direction [29]. For the recellularization of NIH3T3 cells, the cells were effectively aligned with the fiber direction of the HHP-decellularized pericardium compared to the surfactant-decellularized pericardium. This may have been caused by the partially melted and disordered fibers for the surfactant-decellularized pericardium due to the sensitive nature of the fibroblast [30,31]. The cell growth was inhibited on the surfactant-decellularized pericardium compared to the HHP-decellularized pericardium (Figure 7). The residual surfactant in the surfactant-decellularized pericardium probably inhibited the cellular growth because of the high cytotoxicity of SDS [32]. The growth suppression of MSCs on the HHP-decellularized pericardium was also observed, compared to that on TCPS, which suggests that the cellular elongation on the fibrillar structure of the HHP-decellularized pericardium was related to the proliferation [33].

Finally, the recellularized pericardium was rolled up into a cylinder formation and cultivated with the hanging circulation culture method for 2 days (Figure 8). The cylinder formation was maintained without disordering. The cells were still alive in the cylindrically formed pericardium. The cellular features, elongation and alignment, were still maintained on the decellularized pericardium in a cylinder formation, which indicates that the fibrous membranous tissue could contribute to the cellular shape in the tissue, irrespective of the rolling process. These results suggest that the 3D tissue with cells reconstructed by this method could be adapted as a ligament-like tissue, although further studies are needed.

We expect that the proposed tissue-engineered product can be clinically applied. Artificial synthetic grafts and autologous tissue grafts are used in replacement surgery. The deterioration, due to the long-term implantation and loss of function at the site of tissue harvesting, needs to be overcome. In this regard, we proposed a method using tissue engineering techniques. The proposed tissue-engineered product is one of the candidates to overcome the above problems because of its potential for long-term compatibility, owing to the presence of cells inside the tissue. In addition, it may be adapted not only as a graft, but also to the site of harvesting in autologous transplantation. Furthermore, the decellularized pericardium used in this study was flexible, simple to fabricate, and could be processed into various shapes, which could provide various clinical applications, including hand surgery and orthopedic surgery. To widely apply the proposed method, the selection of cells to be recellularized and in vivo studies are necessary.

## 5. Conclusions

We successfully developed a 3D tissue reconstruction method, through which the decellularized pericardium was recellularized and fabricated into a cylinder formation. The cells were elongated and aligned on the decellularized pericardium. The reconstructed decellularized pericardium expressed a mechanical behavior similar to that of porcine ACL. Further in vivo experiments, such as compatibility and mechanical compliance experiments, are needed to apply the 3D-reconstructed decellularized pericardium as an alternative to ligaments and tendons.

## Data Availability

Not applicable.

## Abbreviations

HHP	High hydrostatic pressurization
H&E	Hematoxylin–eosin
SDS	Sodium dodecyl sulfate
SDC	Sodium deoxycholate
FBS	Fatal bovine serum
ACL	Anterior cruciate ligament
TCPS	Tissue culture polystylene

## References

[1] Reing J.E., Brown B.N., Daly K.A., Freund J.M., Gilbert T.W., Hsiong S.X., Huber A., Kullas K.E., Tottey S., Wolf M.T. (2010). The effects of processing methods upon mechanical and biologic properties of porcine dermal extracellular matrix scaffolds. Biomaterials.

[2] Zhu Y., Hideyoshi S., Jiang H.B., Matsumura Y., Dziki J.L., LoPresti S.T., Huleihel L., Faria G.N.F., Fuhrman L.C., Lodono R. (2018). Injectable, Porous, Biohybrid Hydrogels Incorporating Decellularized Tissue Components for Soft Tissue Applications. Acta Biomater..

[3] Andrée B., Bär A., Haverich A., Hilfiker A. (2013). Small Intestinal Submucosa Segments as Matrix for Tissue Engineering: [Review]. Tissue Eng. Part B Rev..

[4] Quinta C., Kondob Y., Mansonc R.L., Lawsonc J.H., Dardikb A., Niklason L.E. (2011). Decellularized tissue-engineered blood vessel as an arterial conduit. Proc. Natl. Acad. Sci. USA.

[5] Lin C.H., Hsia K., Ma H., Lee H., Lu J.H. (2018). In Vivo Performance of Decellularized Vascular Grafts: A Review Article. Int. J. Mol. Sci..

[6] Woods T., Gratzer P.F. (2005). Effectiveness of Three Extraction Techniques in the Development of a Decellularized Bone-Anterior Cruciate Ligament-Bone Graft. Biomaterials.

[7] Jones G., Herbert A., Berry H., Edwards J.H., Fisher J., Ingham E. (2017). Decellularization and Characterization of Porcine Superflexor Tendon: A Potential Anterior Cruciate Ligament Replacement. Tissue Eng. Part A.

[8] Schulze-Tanzil G., Al-Sadi O., Ertel W., Lohan A. (2012). Decellularized Tendon Extracellular Matrix-A Valuable Approach for Tendon Reconstruction?. Cells..

[9] Nakamura N., Kimura T., Kishida A. (2017). Overview of the Development, Applications, and Future Perspectives of Decellularized Tissues and Organs. ACS Biomater. Sci. Eng..

[10] Hashimoto Y., Funamoto S., Sasaki S., Honda T., Hattori S., Nam K., Kimura T., Mochizuki M., Fujisato T., Kobayashi H. (2010). Preparation and Characterization of Decellularized Cornea Using High-Hydrostatic Pressurization for Corneal Tissue Engineering. Biomaterials.

[11] Iablonskii P., Cebotari S., Tudorache I., Granados M., Morticelli L., Goecke T., Klein N., Korossis S., Hilfiker A., Haverich A. (2015). Tissue-Engineered Mitral Valve: Morphology and Biomechanics. Interact. Cardiovasc. Thorac. Surg..

[12] Lu C.C., Zhang T., Amadio P.C., An K.N., Moran S.L., Gingery A., Zhao C. (2019). Lateral Slit Delivery of Bone Marrow Stromal Cells Enhances Regeneration in the Decellularized Allograft Flexor Tendon. J. Orthop. Transl..

[13] Tozer S., Duprez D. (2005). Tendon and Ligament: Development, Repair and Disease. Birth Defects Res. C Embryo Today.

[14] Edgar L., Altamimi A., García Sánchez M.G., Tamburrinia R., Asthana A., Gazia C., Orlando G. (2018). Utility of Extracellular Matrix Powders in Tissue Engineering. Organogenesis.

[15] Ning L.J., Jiang Y.L., Zhang C.H., Zhang Y., Yang J.L., Cui J., Zhang Y.J., Yao X., Luo J.C., Qin T.W. (2017). Fabrication and Characterization of a Decellularized Bovine Tendon Sheet for Tendon Reconstruction. J. Biomed. Mater. Res. A.

[16] Spang M.T., Christman K.L. (2018). Extracellular Matrix Hydrogel Therapies: In Vivo Applications and Development. Acta Biomater..

[17] Pati F., Jang J., Ha D.H., Won Kim S., Rhie J.W., Shim J.H., Kim D.H., Cho D.W. (2014). Printing Three-Dimensional Tissue Analogues with Decellularized Extracellular Matrix Bioink. Nat. Commun..

[18] Badylak S.F., Freytes D.O., Gilbert T.W. (2009). Extracellular Matrix as a Biological Scaffold Material: Structure and Function. Acta Biomater..

[19] Heath D.E. (2019). A Review of Decellularized Extracellular Matrix *Biomaterials* for Regenerative Engineering Applications. Regen. Eng. Transl. Med..

[20] Negishi J., Hashimoto Y., Yamashita A., Zhang Y., Kimura T., Kishida A., Funamoto S. (2017). Evaluation of Small-Diameter Vascular Grafts Reconstructed from Decellularized Aorta Sheets. J. Biomed. Mater. Res. A.

[21] Wu P., Nakamura N., Morita H., Nam K., Fujisato T., Kimura T., Kishida A. (2019). A Hybrid Small-Diameter Tube Fabricated from Decellularized Aortic Intima-Media and Electrospun Fiber for Artificial Small-Diameter Blood Vessel. J. Biomed. Mater. Res. A.

[22] Lichtenberg A., Tudorache I., Cebotari S., Ringes-Lichtenberg S., Sturz G., Hoeffler K., Hurscheler C., Brandes G., Hilfiker A., Haverich A. (2006). In Vitro Re-Endothelialization of Detergent Decellularized Heart Valves under Simulated Physiological Dynamic Conditions. Biomaterials.

[23] Taylor D.A., Sampaio L.C., Ferdous Z., Gobin A.S., Taite L.J. (2018). Decellularized Matrices in Regenerative Medicine. Acta Biomater..

[24] Wu P., Nakamura N., Kimura T., Nam K., Fujisato T., Funamoto S., Higami T., Kishida A. (2015). Decellularized Porcine Aortic Intima-Media as a Potential Cardiovascular Biomaterial. Interact. Cardiovasc. Thorac. Surg..

[25] Laker L., Dohmen P.M., Smit F.E. (2020). Synergy in a Detergent Combination Results in Superior Decellularized Bovine Pericardial Extracellular Matrix Scaffolds. J. Biomed. Mater. Res. A.

[26] Butler D.L., Guan Y., Kay M.D., Cummings J.F., Feder S.M., Levy M.S. (1992). Location-Dependent Variations in the Material Properties of the Anterior Cruciate Ligament. J. Biomech..

[27] Nikkhah M., Eshak N., Zorlutuna P., Annabi N., Castello M., Kim K., Dolatshahi-Pirouz A., Edalat F., Bae H., Yang Y. (2012). Directed Endothelial Cell Morphogenesis in Micropatterned Gelatin Methacrylate Hydrogels. Biomaterials.

[28] Li X., Wang X., Yao D., Jiang J., Guo X., Gao Y., Li Q., Shen C. (2018). Effects of Aligned and Random Fibers with Different Diameter on Cell Behaviors. Colloids Surf. B Biointerfaces.

[29] Humphrey J.D., Dufresne E.R., Schwartz M.A. (2014). Mechanotransduction and Extracellular Matrix Homeostasis. Nat. Rev. Mol. Cell Biol..

[30] Wang B., Shi J., Wei J., Wang L., Tu X., Tang Y., Chen Y. (2017). Fabrication of elastomer pillar arrays with height gradient for cell culture studies. Microelectron. Eng..

[31] Kimura T., Kondo M., Hashimoto Y., Fujisato T., Nakamura N., Kishida A. (2019). Surface Topography of PDMS Replica Transferred from Various Decellularized Aortic Lumens Affects Cellular Orientation. ACS Biomater. Sci. Eng..

[32] Gilbert T.W., Sellaro T.L., Badylak S.F. (2006). Decellularization of Tissues and Organs. Biomaterials.

[33] Xie J., Bao M., Bruekers S.M.C., Huck W.T.S. (2017). Collagen Gels with Different Fibrillar Microarchitectures Elicit Different Cellular Responses. ACS Appl. Mater. Interfaces.

